# Interspinous Spacer Implant in Patients with Lumbar Spinal Stenosis: Preliminary Results of a Multicenter, Randomized, Controlled Trial

**DOI:** 10.1155/2012/823509

**Published:** 2012-02-07

**Authors:** Larry E. Miller, Jon E. Block

**Affiliations:** ^1^Miller Scientific Consulting, Inc., 26 Portobello Road, Arden, NC 28704, USA; ^2^The Jon Block Group, 2210 Jackson Street, Suite 401, San Francisco, CA 94115, USA

## Abstract

A prospective, randomized, controlled trial was conducted to compare clinical outcomes in patients treated with an investigational interspinous spacer (Superion) versus those treated with an FDA-approved spacer (X-STOP). One hundred sixty-six patients with moderate lumbar spinal stenosis (LSS) unresponsive to conservative care were treated randomly with the Superion (*n* = 80) or X-STOP (*n* = 86) interspinous spacer. Study subjects were followed through 6 months posttreatment. Zurich Claudication Questionnaire (ZCQ) symptom severity scores improved 30% with Superion and 25% with X-STOP (both *P* < 0.001). Similar changes were noted in ZCQ physical function with improvements of 32% with Superion and 27% with X-STOP (both *P* < 0.001). Mean ZCQ patient satisfaction score ranged from 1.7 to 2.0 in both groups at all follow-up visits. The proportion of subjects that achieved at least two of three ZCQ clinical success criteria at 6 months was 75% with Superion and 67% with X-STOP. Axial pain decreased from 55 ± 27 mm at pretreatment to 22 ± 26 mm at 6 months in the Superion group (*P* < 0.001) and from 54 ± 29 mm to 32 ± 31 mm with X-STOP (*P* < 0.001). Extremity pain decreased from 61 ± 26 mm at pretreatment to 18 ± 27 mm at 6 months in the Superion group (*P* < 0.001) and from 64 ± 26 mm to 22 ± 30 mm with X-STOP (*P* < 0.001). Back function improved from 38 ± 13% to 21 ± 19% with Superion (*P* < 0.001) and from 40 ± 13% to 25 ± 16% with X-STOP (*P* < 0.001). Preliminary results suggest that the Superion interspinous spacer and the X-STOP each effectively alleviate pain and improve back function in patients with moderate LSS who are unresponsive to conservative care.

## 1. Introduction

Lumbar spinal stenosis (LSS) is defined as a narrowing of the lumbar spinal canal and/or the intervertebral foramina due to disc degeneration, bulging of the annulus, facet joint hypertrophy, and/or thickening of the ligamentum flavum [1–3]. This progressive narrowing causes compression of the neurovascular structures in the lumbar spine [4], which manifests as intermittent neurologic symptoms (typically low back and leg pain) with standing, ambulating, and trunk extension. The annual incidence of LSS is 5 per 100,000 people [5], and, in adults over the age of 65 years, LSS is the most common diagnosis in those who undergo spine surgery [6].

Nonsurgical management options such as activity modification, physical therapy, anti-inflammatory drugs, and epidural steroid injections represent the standard of care for initial treatment of mild claudication symptoms. The long-term effectiveness of these nonsurgical treatments is limited, however, since none hinders the progression of the disease [7–9]. This is supported by the fact that only 42% of patients treated with nonsurgical management for LSS report improvement in symptoms 10 years later [10].

Decompression surgery is often recommended to patients with severe claudication symptoms who fail conservative care. In fact, surgery is performed on 21% of patients within 3 years of an LSS diagnosis [11]. However, the decision of if and when to operate varies widely among physicians and is influenced by physician preference more so than disease characteristics [12]. Consequently, many patients may unnecessarily undergo invasive decompression surgery (with or without fusion) for less-than-severe claudication symptoms, a procedure associated with significant cost and morbidity [6, 13–15].

Implantation with a minimally invasive interspinous spacer is an alternative treatment option for patients with mild to moderate neurogenic intermittent claudication secondary to LSS. An interspinous spacer is implanted between contiguous spinous processes and acts to limit back extension at the symptomatic level. The rationale for the utility of these devices is that low back pain secondary to LSS is often alleviated when patients flex their lumbar spine and is exacerbated with spinal extension [16]. Although use of interspinous spacers dates back to the 1950s, frequent dislodgements caused these devices to largely fall out of favor until the last decade. Midterm results with newer devices suggest that interspinous spacers improve patient symptoms with rare reports of dislodgements [17, 18], although long-term safety and effectiveness are unknown [19]. The purpose of this prospective, randomized, controlled trial was to compare 6-month clinical outcomes in patients treated with an investigational interspinous spacer versus those treated with a Food and Drug Administration- (FDA-) approved spacer.

## 2. Methods

### 2.1. Clinical Trial Overview

This prospective, multicenter, randomized, controlled, FDA-IDE trial enrolled 166 patients (80 Superion, 86 X-STOP) from 22 sites in the United States from September 2008 to December 2010. All research procedures performed in this trial were in strict accordance with a predefined protocol that was approved by all researchers and the institutional review board at each respective site. The procedures used in this clinical trial were in accordance with the recommendations of the Helsinki Declaration, and each patient gave written, informed consent before surgery. This trial is registered under ClinicalTrials.gov number NCT00692276.

### 2.2. Subjects

Inclusion criteria for this trial included (a) age ≥45 years, (b) persistent leg, buttock, or groin pain, with or without back pain, which was relieved by lumbar flexion, (c) symptomatic and undergoing unsuccessful conservative treatment for at least 6 months, (d) diagnosis of moderate LSS, defined as 25% to 50% reduction in lateral/central foramen diameter compared to adjacent levels, and radiographic evidence of thecal sac and/or cauda equine compression, nerve root impingement by either osseous or nonosseous elements, and/or hypertrophic facets with canal encroachment, (e) Zurich Claudication Questionnaire Physical Function score ≥2.0, (f) able to sit for 50 minutes without pain and to walk ≥50 feet, and (g) able to provide voluntary informed consent and to comply with the study procedures.

Exclusion criteria included (a) LSS at three or more levels, (b) concomitant surgical procedure required, (c) grade II to V spondylolisthesis, (d) unremitting back pain in any spinal position, (e) significant lumbar instability, defined by ≥3 mm translation or ≥5° angulation, (f) active systemic disease that may affect the welfare of the patient, (g) vertebral osteoporosis or history of vertebral fracture, (h) body mass index ≥40 kg/m^2^, (i) previous lumbar spine surgery, (j) pregnant or lactating female, and (k) any disease or condition that, in the investigator's opinion, may affect subject safety or confound trial outcomes.

### 2.3. Devices

**Figure 1:**
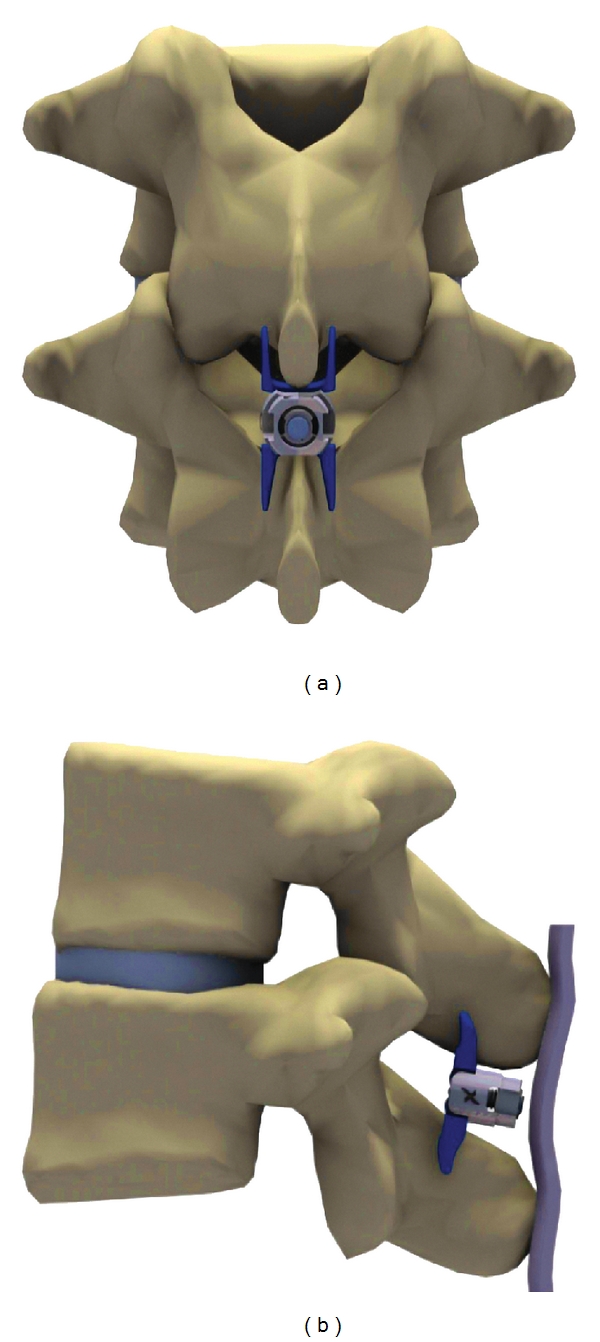
Superion interspinous spacer *in situ*. (a) A/P view, (b) lateral view.

Subjects were treated randomly with the superion interspinous spacer system (Vertiflex, Inc., San Clemente, CA, USA) or the X-STOP Interspinous Process Decompression System (Medtronic, Inc., Sunnyvale, CA, USA). The Superion device (Figures 1(a) and 1(b)) is an investigational device under study in this FDA-approved IDE clinical trial, which is composed of titanium 6AI-4 V ELI alloy, a material commonly used in a variety of orthopedic applications that conforms to ASTM standards for surgical implants [20]. Five device sizes are available, ranging from 8 to 16 mm, with the sizes corresponding to the amount of desired distraction between the two spinous processes. This single-piece, self-expanding implant is percutaneously delivered and deployed between the spinous processes of the symptomatic vertebral levels.

The X-STOP device was approved for use in the United States by the FDA in November 2005 and served as the control device in this trial. The X-STOP device has been described in detail elsewhere [21].

### 2.4. Pretreatment Procedures

Baseline assessments included a complete physical examination, medical history, and study eligibility based on the inclusion/exclusion criteria. Preprocedural radiographic assessments included X-rays (standing A/P, lateral lumbar, flexion/extension lateral lumbar) and magnetic resonance imaging or computed tomography of the lumbar spine. The Zurich Claudication Questionnaire (ZCQ) was utilized to assess patient-reported measures of symptom severity, physical function, and patient satisfaction [22]. Extremity and axial pain severity were measured with a 100 mm visual analogue scale. Degree of back-specific functional disability was assessed with the Oswestry Disability Index (ODI) (version 2) on a 0 to 100% scale [23].

### 2.5. Treatment

**Figure 2:**
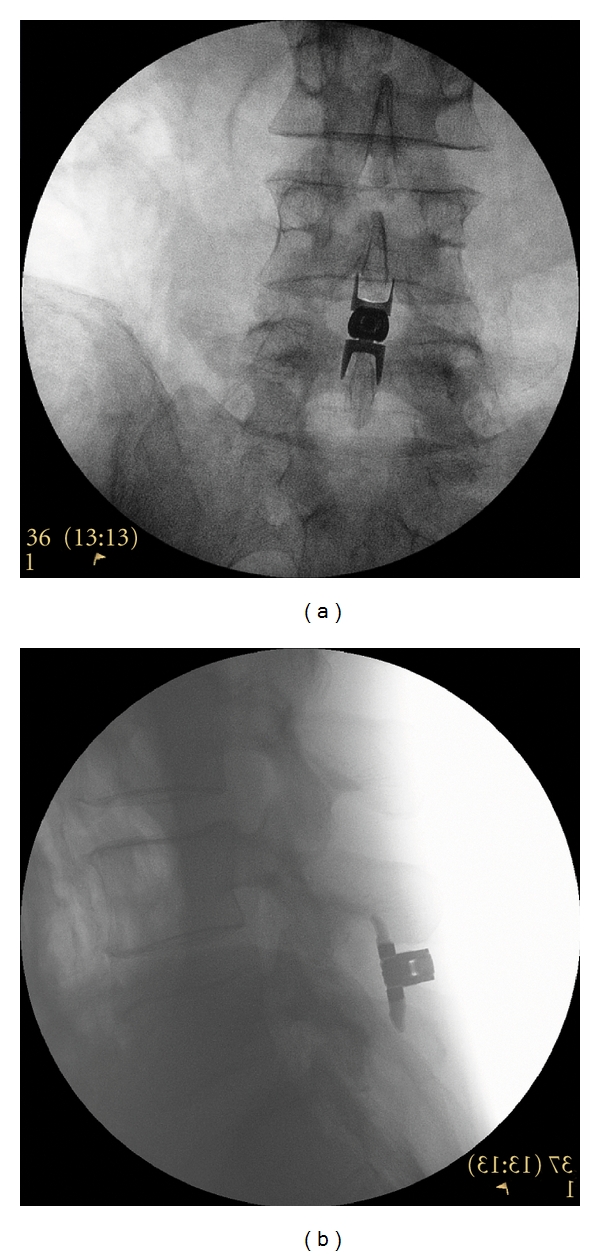
(a) A/P and (b) lateral radiographic image showing a properly placed Superion Interspinous Spacer.

Despite minor differences in the surgical technique for insertion of these devices, the procedures are quite similar and are collectively described hereafter. Subjects were laid prone on a radiolucent table with the lumbar spine in a neutral or slightly flexed position. Under fluoroscopic guidance or direct visualization, the surgical level was identified and a midline incision was made (Superion: 24 ± 11 mm, X-STOP: 54 ± 21 mm, *P* < 0.001). The supraspinous ligament was longitudinally dissected at the symptomatic level and then dilated to ensure adequate room to maneuver within the interspinous space. A cannula was inserted over the dilator and proper alignment and depth were ensured before dilator removal. Next, an interspinous gauge was inserted through the cannula to determine proper implant size selection, and final midline positioning was confirmed under fluoroscopy. The appropriately sized spacer was delivered through the cannula using a device inserter that loaded, inserted into the interspinous space via the cannula, and deployed the implant. Proper device placement was confirmed with fluoroscopy. Finally, the inserter and cannula were removed, and the incision was sutured in a standard fashion. Proper postoperative placement of the Superion device is illustrated radiographically in Figures 2(a) and 2(b).

### 2.6. Followup

Postoperatively, subjects were followed through discharge and returned for visits at 6 weeks, 3 months, and 6 months. Radiographic evaluations included standing A/P, lateral lumbar, and flexion/extension lateral lumbar (the latter was not performed at discharge) X-rays. The ZCQ, extremity and axial pain severity, and back-specific functional disability were assessed at 6 weeks, 3 months, and 6 months. The FDA-mandated primary endpoint of this IDE clinical trial is 2 years, with postmarket surveillance scheduled for 10 years.

### 2.7. Sample Size

The sample size for this noninferiority trial was estimated by assuming a 2-year overall success rate of 62.5% with the Superion device, a 60% success rate with the X-STOP device, 90% statistical power, and a noninferiority margin of 10%. Based on these assumptions, 300 to 600 total subjects (depending on enrollment time) were required using a Bayesian adaptive approach. This paper describes the preliminary outcomes of the first 166 subjects enrolled in this clinical trial.

### 2.8. Randomization and Blinding

Randomization was achieved by using a computer-generated master randomization list of treatment assignments using a 1 : 1 allocation. Sites utilized a web-based system to obtain randomization assignment before each subject was enrolled. Treatments were not concealed to investigators, outcome assessors, or trial participants.

### 2.9. Data Analysis

Data were analyzed using Predictive Analytics Software (v. 18, SPSS, Inc., Chicago, IL). Continuous data were reported as mean ± SD and categorical data were reported as frequencies and percentages. Longitudinal changes in clinical outcomes were assessed with two-way (time-by-treatment) repeated measures analysis of variance. Area under the curve (AUC) was calculated for pain severity and back function scores according to the methodology of Matthews et al. [24]. Briefly, this analysis uses all responses of each subject to calculate a single number that summarizes the response curve, after accounting for differential followup intervals among subjects. Clinical success was defined as a ≥0.5 point improvement in ZCQ symptom severity and physical function [22], ZCQ patient satisfaction score ≤2.5 [22], ≥20 mm improvement in pain scores [25, 26] and a ≥15 percentage point improvement in ODI [25, 27], respectively.

## 3. Results

### 3.1. Subject Flow

**Figure 3:**
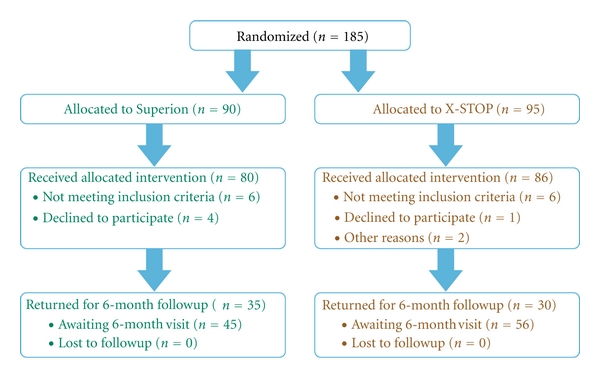
Patient flow diagram.

A total of 185 subjects were randomized in this clinical trial—90 to Superion and 95 to X-STOP (Figure 3). Approximately 10% of randomized subjects were withdrawn from the study before treatment due to not meeting inclusion criteria during this period (*n* = 12), declining to participate (*n* = 5), or other reasons (*n* = 2). Ultimately, 80 subjects were treated with the Superion device and 86 were treated with X-STOP. At the time of this analysis, 66 subjects (36 Superion, 30 X-STOP) had passed the 6-month followup visit window.

### 3.2. Subject Characteristics

Baseline subject characteristics (Table 1), device utilization, procedure time, and procedural blood loss (Table 2) were comparable between groups.

### 3.3. Zurich Claudication Questionnaire

**Figure 4:**
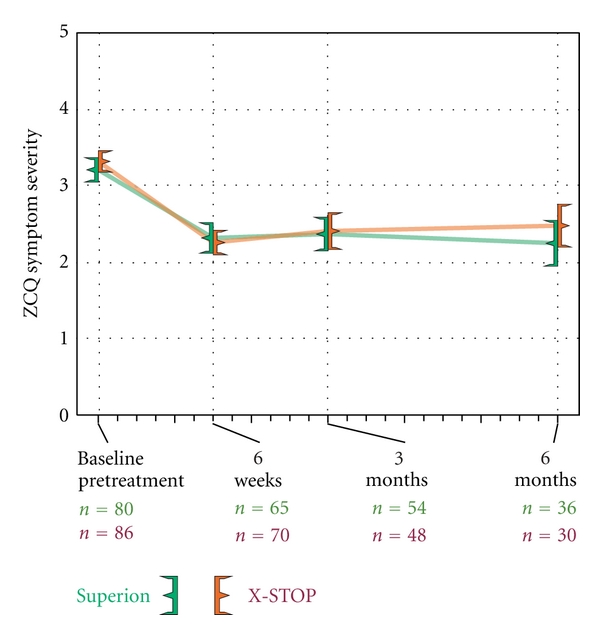
Improvement in ZCQ symptom severity scores through 6 months posttreatment. Values are mean ± 95% CI.

**Figure 5:**
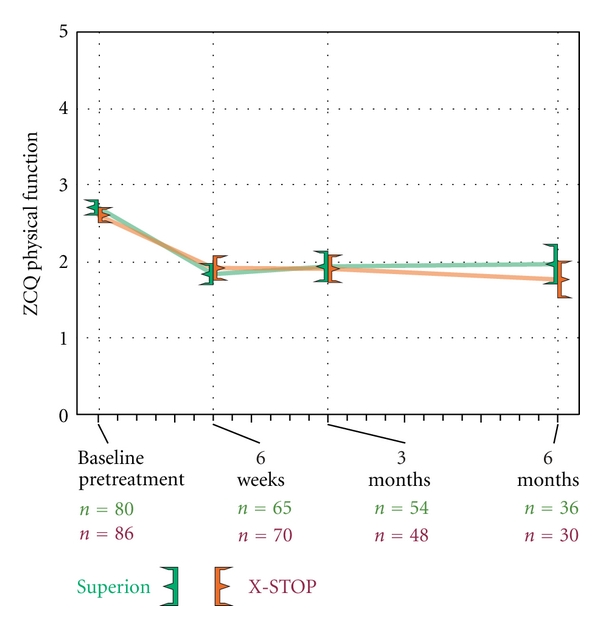
Improvement in ZCQ physical function scores through 6 months posttreatment. Values are mean ± 95% CI.

**Figure 6:**
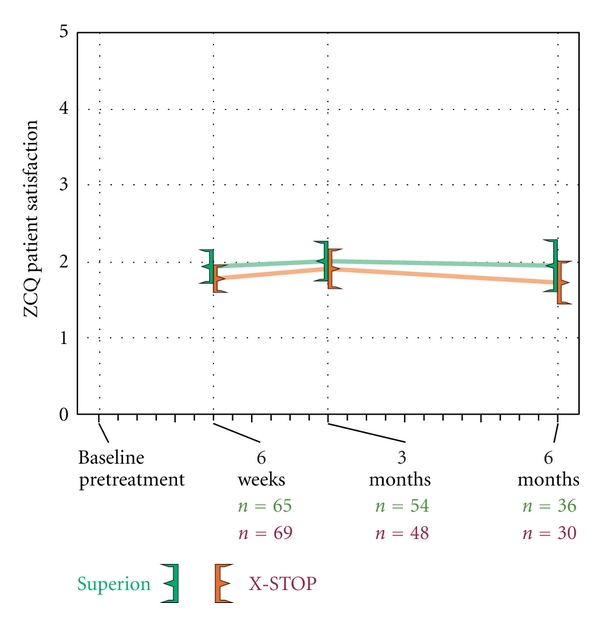
ZCQ patient satisfaction scores through 6 months posttreatment. Values are mean ± 95% CI.

**Figure 7:**
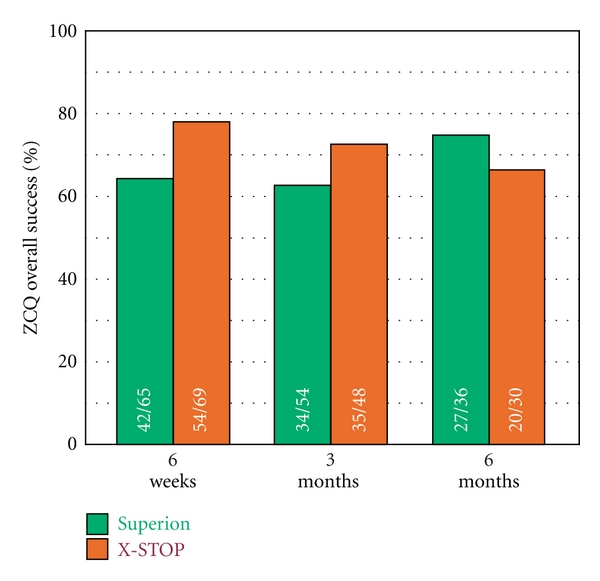
Overall ZCQ success rates through 6 months posttreatment. Values are mean ± 95% CI.

ZCQ symptom severity scores improved 30% with Superion and 25% with X-STOP through 6 months (both *P* < 0.001) (Figure 4). Similar changes were noted in ZCQ physical function with improvements of 32% with Superion and 27% with X-STOP through 6 months (both *P* < 0.001) (Figure 5). The mean ZCQ patient satisfaction score ranged from 1.7 to 2.0 in both groups at all followup visits (Figure 6). The proportion of subjects who achieved ZCQ clinical success with Superion and X-STOP was 75% and 53% for symptom severity, 64% and 63% for physical function, and 78% and 93% for patient satisfaction. The proportion of subjects that achieved at least two of three ZCQ clinical success criteria at 6 months was 75% with Superion and 67% with X-STOP (Figure 7).

### 3.4. Axial Pain Severity

**Figure 8:**
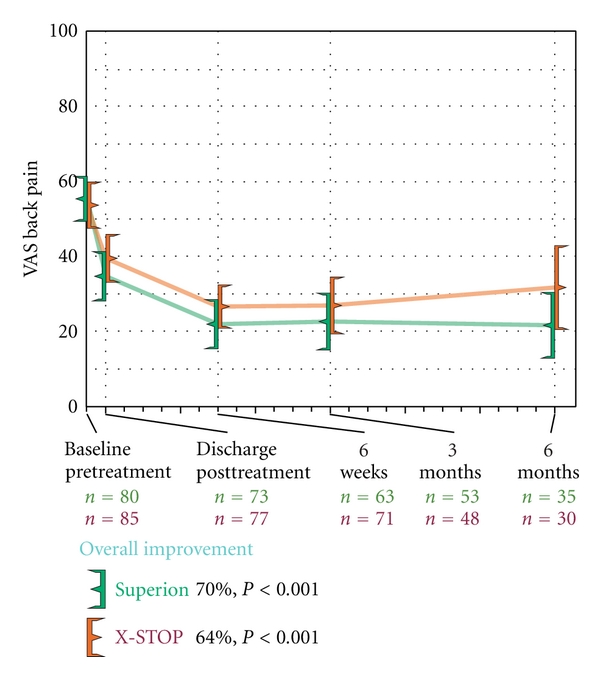
Improvement in axial pain severity through 6 months posttreatment. Values are mean ± 95% CI mm.

**Figure 9:**
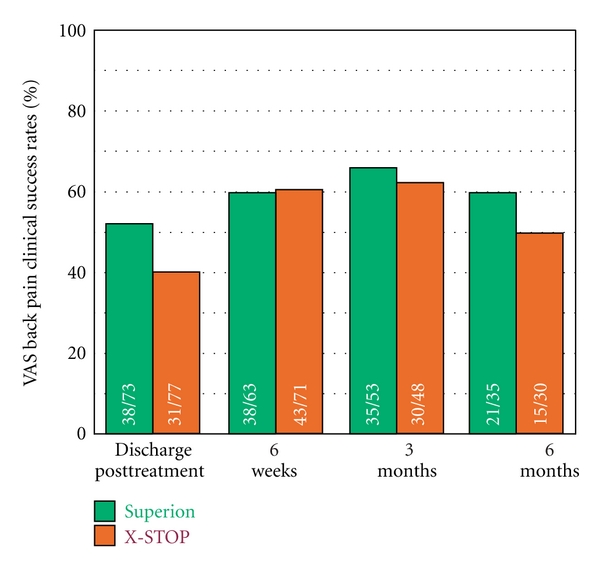
Axial pain severity clinical success rates through 6 months posttreatment. Clinical success defined as a ≥20 mm improvement from pretreatment.

**Figure 10:**
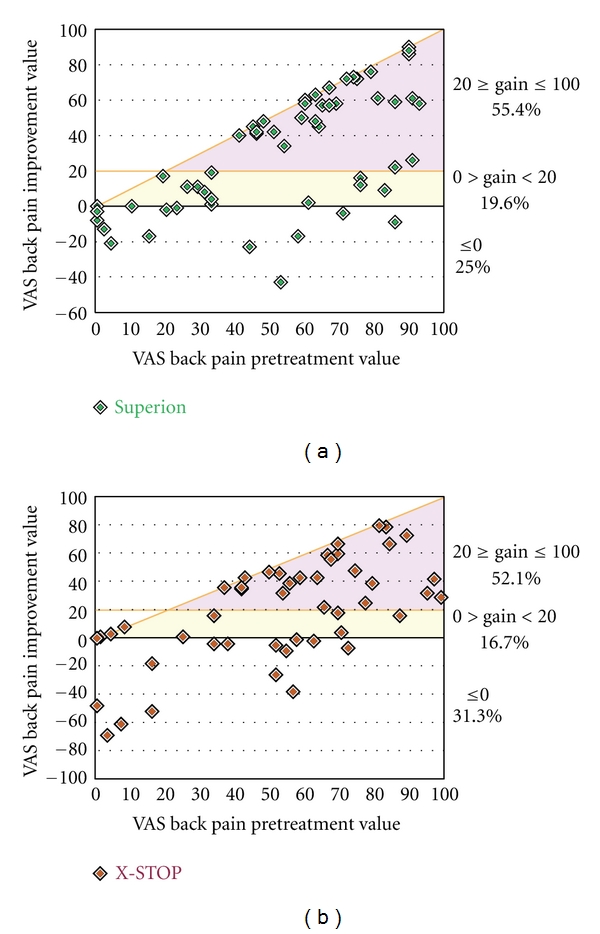
Relationship of pretreatment axial pain severity with absolute axial pain improvement at 6 months. (a) Superion, (b) X-STOP. Data are reported in mm and include values from pretreatment and from the final subject followup visit at or beyond 3 months. The horizontal line represents the absolute change threshold (20 mm) for clinical success. Subjects on or above this line achieved clinical success. The diagonal line represents the maximum achievable absolute improvement based on the pretreatment value.

Axial pain decreased from 55 ± 27 mm at pretreatment to 22 ± 26 mm at 6 months in the Superion group, which represented a 70% median improvement compared to pretreatment values (*P* < 0.001). Subjects treated with the X-STOP device realized axial pain median improvements of 64% (*P* < 0.001), from 54 ± 29 mm at pretreatment to 32 ± 31 mm at 6 months (Figure 8). Axial pain severity AUC across the entire range of subject followup was 29 ± 21 mm for Superion and 37 ± 22 mm for X-STOP. At 6 months posttreatment, 60% (21 of 35) of Superion subjects and 50% (15 of 30) of X-STOP subjects achieved axial pain clinical success (Figure 9). A strong positive relationship was noted between pretreatment axial pain severity and magnitude of improvement following interspinous spacer treatment in both groups (Figures 10(a) and 10(b)).

### 3.5. Extremity Pain Severity

**Figure 11:**
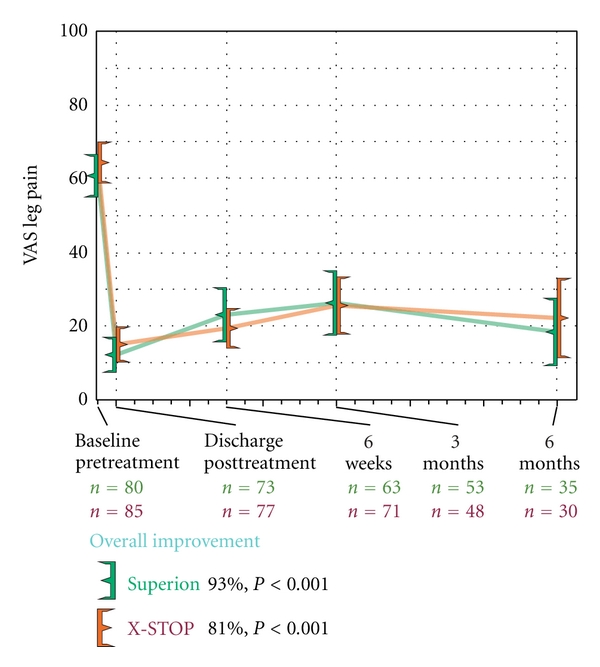
Improvement in extremity pain severity through 6 months posttreatment. Values are mean ± 95% CI mm.

**Figure 12:**
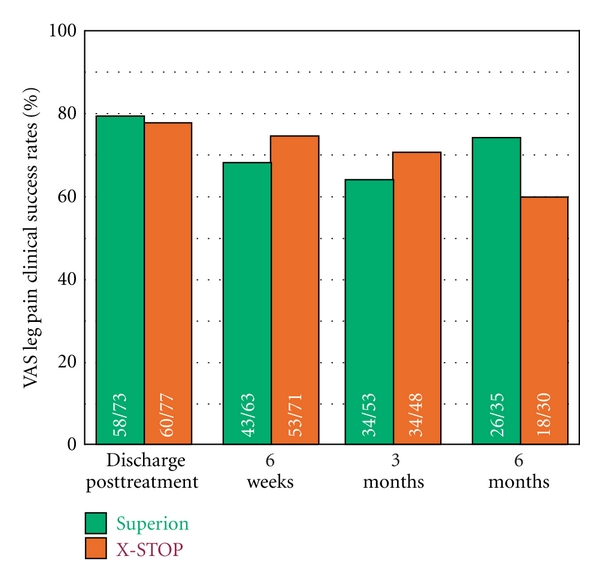
Extremity pain severity clinical success rates through 6 months posttreatment. Clinical success defined as a ≥20 mm improvement from pretreatment.

**Figure 13:**
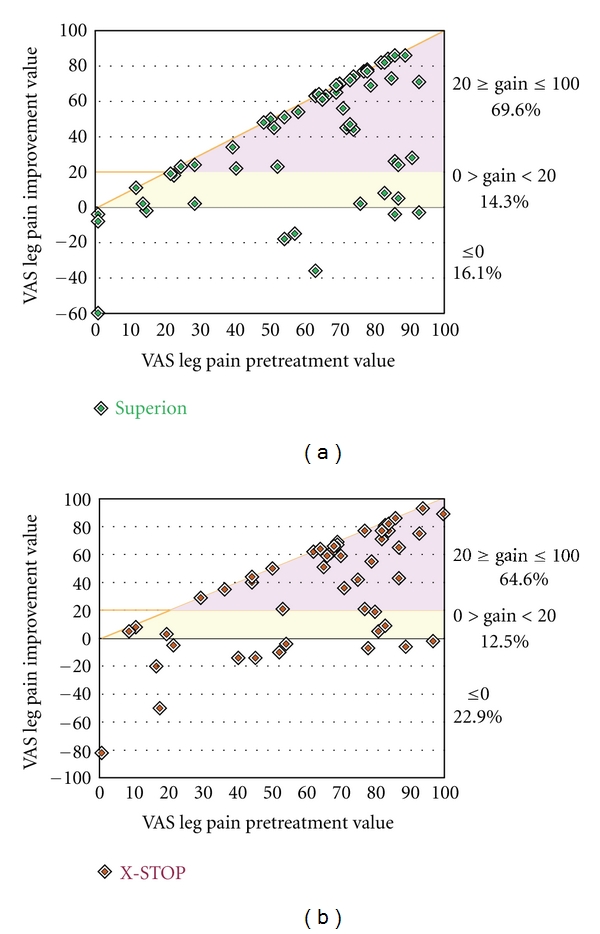
Relationship of pretreatment extremity pain severity with absolute extremity pain improvement at 6 months. (a) Superion, (b) X-STOP. Data are reported in mm and include values from pretreatment and from the final subject followup visit at or beyond 3 months. The horizontal line represents the absolute change threshold (20 mm) for clinical success. Subjects on or above this line achieved clinical success. The diagonal line represents the maximum achievable absolute improvement based on the pretreatment value.

Both treatment groups demonstrated statistically significant (*P* < 0.001) reductions in extremity pain severity through 6 months posttreatment. Superion subjects improved from a score of 61 ± 26 mm at pretreatment to 18 ± 27 mm at 6 months, a 93% median decrease. Subjects treated with the X-STOP device improved from 64 ± 26 mm at pretreatment to 22 ± 30 mm at 6 months, an 81% median decrease (Figure 11). The extremity pain severity AUC across the entire range of subject followup was 31 ± 23 mm for Superion versus 34 ± 21 mm for X-STOP. At 6 months posttreatment, 74% (26 of 35) of Superion subjects and 60% (18 of 30) of X-STOP subjects achieved extremity pain clinical success (Figure 12). A strong positive relationship was noted between pretreatment extremity pain severity and magnitude of improvement following interspinous spacer treatment in both groups (Figures 13(a) and 13(b)).

### 3.6. Back-Specific Functional Impairment

**Figure 14:**
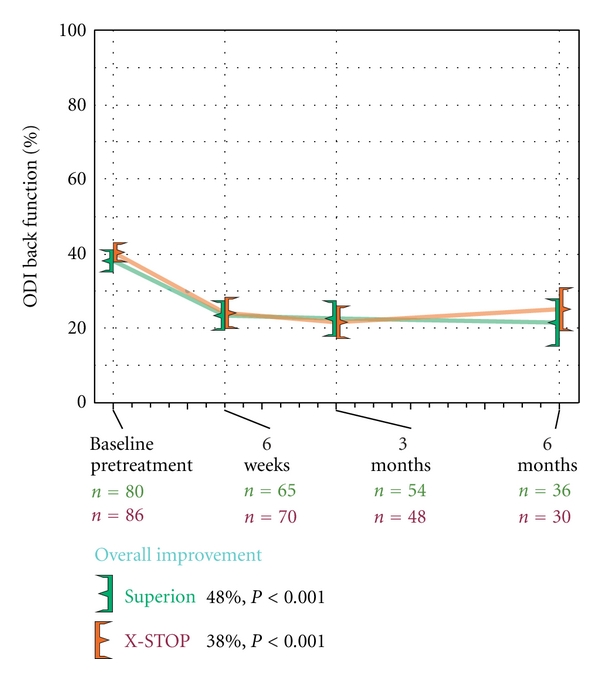
Improvement in back function through 6 months posttreatment. Values are mean ± 95% CI.

**Figure 15:**
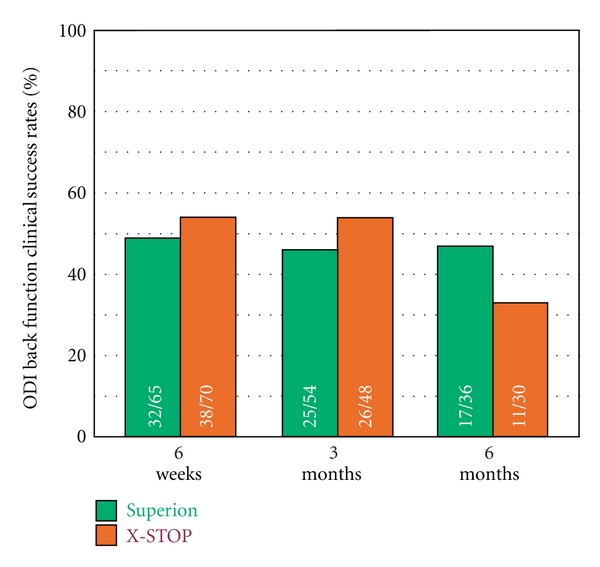
Back function clinical success rates through 6 months posttreatment. Clinical success defined as a ≥15 percentage point improvement from pretreatment.

**Figure 16:**
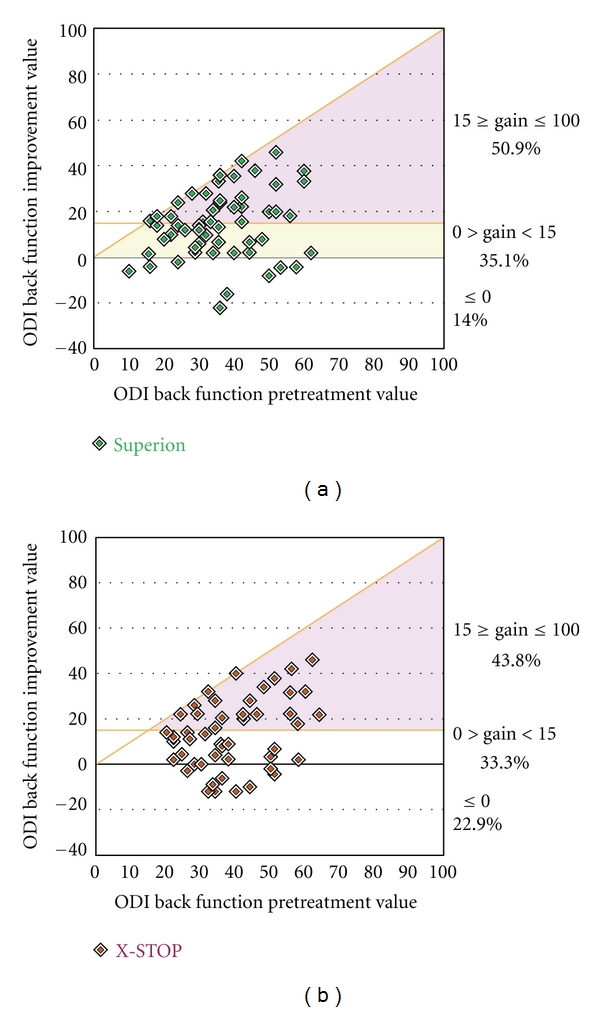
Relationship of pretreatment back function with absolute back function improvement at 6 months. (a) Superion, (b) X-STOP. Data are reported as percentages and include values from pretreatment and from the final subject followup visit at or beyond 3 months. The horizontal line represents the absolute change threshold (15 percentage points) for clinical success. Subjects on or above this line achieved clinical success. The diagonal line represents the maximum achievable absolute improvement based on the pretreatment value.

Back function improved a median of 48% (*P* < 0.001) through 6 months posttreatment with the Superion device (38 ± 13% to 21 ± 19%) while improvements were 38% (40 ± 13% to 25 ± 16%) with X-STOP (*P* < 0.001) (Figure 14). The back function AUC across the entire range of subject followup was 26 ± 14% for Superion versus 29 ± 14% for X-STOP. Back function clinical success was achieved in 47% (17 of 36) with Superion and 37% (11 of 30) with X-STOP (Figure 15). No relationship was noted between pretreatment back function and magnitude of improvement following interspinous spacer treatment in either group (Figures 16(a) and 16(b)).

### 3.7. Complications

Device-related complications were note in 7 patients per treatment group through 6 months followup. In patients treated with the Superion device, 4 explants were performed between 1 and 4 months posttreatment due to persistent pain. Two revisions were performed—a foraminotomy at 4 months and a left-side decompression at 5 months with the Superion device left *in situ*. Spinal process fracture was noted in one subject.

In patients treated with the X-STOP, two explants were performed following device dislodgement at 1 month and evidence of lumbosacral disc herniation at 4 months, respectively. Spinal process fracture was noted in three subjects, wound infection requiring readmission in one subject, and a nerve root block for persistent pain at 4 months.

## 4. Discussion

Short-term clinical outcomes following implantation with the Superion interspinous spacer system demonstrate amelioration of low back and leg pain and improvements in back function in patients with LSS who are unresponsive to conservative treatments. The Superion device is a promising therapeutic option in patients with moderate LSS with 6-month clinical outcomes similar to those achieved with the FDA-approved X-STOP device.

The clinical outcomes with the Superion device in this clinical trial are comparable to those reported in the study of Bini and colleagues who reported 1-year outcomes of 52 patients treated with the Superion device for moderate LSS [28]. In that study, axial pain severity improved from 6.9 ± 1.1 at pretreatment to 3.4 ± 1.5 at 1 year, which represented a 49% improvement (*P* < 0.001). Extremity pain severity similarly decreased from 6.6 ± 1.4 at pretreatment to 2.8 ± 1.5 at 1 year, reflecting a 53% overall improvement (*P* < 0.001). Back function improved from 60 ± 8% at pretreatment to 21 ± 14% at 1 year, representing a 64% (*P* < 0.001) improvement. For comparison, median percent improvements 6 months following implantation with the Superion device in the current trial were 70%, 93%, and 48% for axial pain, extremity pain, and back function, respectively.

Despite significant pain relief following implantation with the Superion device, the mode of action with interspinous spacers remains controversial. Potential mechanisms include widening of the lumbar spinal canal and increases in disc heights via indirect decompression and reductions in intervertebral mobility although these have not been reliably demonstrated. A cadaver study demonstrated that implantation of the Superion device prevents supraphysiological motion at the symptomatic level and has no adverse impact on the local anatomy [29]. However, given the weak relationship between radiographic findings and patient symptoms in LSS, the clinical importance of this finding is unknown.

A limitation of this clinical trial was the relatively short-term followup period. Subjects in this trial will, however, continue to be followed through 10 years posttreatment. In addition, these preliminary results should be interpreted with caution since total enrollment is anticipated to exceed 300 subjects. Blinding was not utilized in this trial and, therefore, the potential for ascertainment bias should be considered. Importantly, interspinous spacers are appropriate only for patients that meet strict clinical and radiographic criteria. Finally, we report no radiographic range of motion data and, therefore, cannot comment on the mechanism of improvement observed in these patients.

In conclusion, the Superion and X-STOP interspinous spacers effectively ameliorate pain and improve back function through 6 months in carefully selected patients with moderate LSS who are unresponsive to conservative care.

## Figures and Tables

**Table 1:** Baseline patient characteristics.

Characteristic	Superion (*n* = 80)	X-STOP (*n* = 86)
*Age*, mean ± SD*, y *	67 ± 9	67 ± 11
*Male*, *n* (%)	**48** (60)	**57** (66)
*Body mass index*, mean ± SD, kg/m^2^	30 ± 5	30 ± 5

*Tobacco use*, *n (%) *		
None	**34** (43)	**41** (48)
Previous use	**35** (44)	**34** (40)
Current use	**11** (14)	**11** (13)
*Axial pain severity score*, mean ± SD, mm	55 ± 27	54 ± 29
*Extremity pain severity score*, mean ± SD, mm	61 ± 26	64 ± 26
*Oswestry disability index*, mean ± SD, %	38 ± 13	40 ± 13

**Table 2:** Procedural data.

Characteristic	Superion (*n* = 80)	X-Stop (*n* = 86)
Anesthesia, *n* (%)*		
General	**68 ** (85)	**79 ** (92)
Conscious sedation	**12** (15)	**7** (8)
Local	**7**(9)	**4** (5)

Number of devices, *n* (%)		
One	**40** (50)	**38** (44)
Two	**40** (50)	**48** (56)

Device size, *n* (%)		
6 mm	**NA**	**2** (2)
8 mm	**1** (1)	**5** (4)
10 mm	**15** (12)	**38** (28)
12 mm	**32** (27)	**51** (38)
14 mm	**63** (52)	**34** (25)
16 mm	**9** (8)	**NA**
NR	**0** (—)	**4** (3)

*Procedure time*, median, mins	55	45
*Procedural blood loss*, median, cc	10	25

*The sum of percentages is greater than 100% because more than one anesthesia type was used in some patients. NR, not reported. NA, not applicable.
